# Metronidazole-Induced Encephalopathy in a Patient With Hepatitis B Cirrhosis During Treatment for Intra-abdominal Abscess

**DOI:** 10.7759/cureus.97786

**Published:** 2025-11-25

**Authors:** Ayaka Takahashi, Masamichi Kimura, Koji Nishikawa, Jun Imamura, Kiminori Kimura

**Affiliations:** 1 Hepatology, Tokyo Metropolitan Cancer and Infectious Diseases Center Komagome Hospital, Tokyo, JPN

**Keywords:** cirrhosis, dentate nucleus, diffusion-weighted imaging, encephalopathy, metronidazole

## Abstract

Metronidazole-induced encephalopathy is an uncommon but potentially reversible neurotoxicity characterized by symmetric dentate-nucleus hyperintensities on magnetic resonance imaging. Reduced drug clearance in cirrhosis may lower the toxicity threshold. We report a 72-year-old man with hepatitis B virus-related cirrhosis and hepatocellular carcinoma who underwent right hemicolectomy for bowel perforation due to peritoneal dissemination and subsequently developed recurrent intra-abdominal/retroperitoneal abscesses, with *Bacteroides thetaiotaomicron* isolated from drainage. Metronidazole 1,500 mg/day was initiated (day 0), briefly substituted with an alternative agent because the patient experienced nausea, and then reintroduced, after which the patient developed dizziness followed by dysarthria and cerebellar ataxia. Brain magnetic resonance imaging on day 63 revealed symmetric T2-weighted and fluid-attenuated inversion recovery hyperintensities in both dentate nuclei consistent with metronidazole-induced encephalopathy. Discontinuation of metronidazole resulted in a gradual clinical improvement, and follow-up magnetic resonance imaging on day 92 showed complete resolution, with a cumulative dose of 76.5 g. This case highlights the occurrence of metronidazole-induced encephalopathy at a sub-100-g exposure in cirrhosis. Thus, new cerebellar signs during therapy should prompt immediate withdrawal, early magnetic resonance imaging, and avoidance of re-exposure.

## Introduction

Metronidazole is widely prescribed for anaerobic and protozoal infections across gastroenterology, hepatology, and surgical practice because of its favorable oral bioavailability, tissue penetration, and low cost; however, although uncommon, metronidazole-induced encephalopathy (MIE) is a clinically important adverse event that may be overlooked when neurological concerns are attributed to an underlying illness or postoperative deconditioning [1-3]. Clinically, MIE often presents with a subacute cerebellar syndrome, gait ataxia, limb dysmetria, and dysarthria, sometimes accompanied by vestibular symptoms, distal paresthesia, or altered mentation; radiologically, the hallmark is bilateral dentate-nucleus hyperintensity on T2-weighted (T2) and fluid-attenuated inversion recovery (FLAIR) with frequent involvement of the dorsal brainstem tegmentum and splenium of the corpus callosum [4,5]. Diffusion-weighted imaging (DWI) typically shows hyperintensity with normal-to-increased apparent diffusion coefficients (ADCs), supporting a predominantly vasogenic process, although cytotoxic-appearing lesions are reported in severe cases that may correlate with a slower recovery [2,3,6].

Time-to-onset and cumulative dose vary widely: although toxicity has been classically linked to prolonged exposure, MIE can emerge after relatively short treatment courses and at cumulative doses <100 g, indicating substantial inter-individual variability in susceptibility [7]. Patient-level risk factors repeatedly cited in case series and reviews include hepatic or renal dysfunction, metabolic derangements and alcohol use, malnutrition, prolonged therapy, and, importantly, re-exposure after a previous adverse neurological episode [3,7,8]. Among these, cirrhosis is particularly relevant to hepatology practice, as impaired hepatic reserve reduces systemic clearance and extends the half-life of metronidazole and its metabolites, plausibly increasing central nervous system exposure and lowering the clinical threshold for neurotoxicity [4,5,9]. Beyond case literature, pharmacoepidemiologic analyses suggest a small but measurable increase in the odds of central and peripheral neurological events after a recent metronidazole exposure, reinforcing the need for vigilance during prolonged anaerobic coverage in older adults or those with organ dysfunction [7,10,11].

Against this backdrop, we report MIE in a hepatitis B virus-related cirrhotic patient treated for recurrent intra-abdominal abscesses, highlighting rapid symptom evolution after a rechallenge, complete radiologic reversibility after drug cessation, and considerations specific to hepatology settings where metronidazole is frequently prescribed.

## Case presentation

A 72-year-old man with hepatitis B virus-related cirrhosis and recurrent hepatocellular carcinoma treated over the years with surgical resection, radiofrequency ablation, transarterial chemoembolization, targeted agents, and immunotherapy underwent right hemicolectomy for bowel perforation due to peritoneal dissemination. He subsequently developed recurrent intra-abdominal and retroperitoneal abscesses, and the drainage culture yielded *Bacteroides thetaiotaomicron*. Metronidazole 1,500 mg/day (500 mg three times daily) was initiated (day 0).

On day 31, he developed nausea; metronidazole was discontinued on day 35 and switched to clindamycin. However, new-onset liver dysfunction subsequently emerged during clindamycin therapy, and metronidazole was reintroduced at the same dose on day 47. Dizziness appeared on day 52, followed by progressive dysarthria and gait/limb ataxia on day 54. He was briefly discharged on day 61, although he returned with worsening gait impairment and anorexia.

On readmission, the patient's vital signs were as follows: body temperature 36.1°C, blood pressure 91/67 mmHg, heart rate 128/min (sinus tachycardia), respiratory rate 19/min, and oxygen saturation 97% on room air. A neurological examination revealed dysarthria, limb ataxia, decreased deep tendon reflexes, and distal paresthesia with preserved superficial sensation. Twelve-lead electrocardiography showed sinus tachycardia with complete right bundle branch block; transthoracic echocardiography results were unremarkable. Abdominal ultrasonography demonstrated no intestinal dilatation or wall thickening and no new hepatobiliary abnormalities.

Laboratory test results at admission indicated no hyperammonemia or hyponatremia (ammonia 55 µg/dL; sodium 137 mmol/L), arguing against metabolic/hepatic encephalopathy or sodium-related encephalopathy as primary drivers. Renal function was preserved (serum creatinine 0.84 mg/dL), and systemic inflammation was low (C-reactive protein 0.27 mg/dL) (Table 1). Serial hematologic and biochemical parameters showed no clinically meaningful change between the index admission and the readmission.

**Table 1:** Results of the laboratory blood examination at admission Alb: albumin; ALT: alanine aminotransferase; ALP: alkaline phosphatase; AST: aspartate aminotransferase; BUN: blood urea nitrogen; Cre: creatinine; CRP: C-reactive protein; γ-GTP: gamma-glutamyl transpeptidase; Hb: hemoglobin; HBcAb: hepatitis B core antibody; HBsAg: hepatitis B surface antigen; HBsAb: hepatitis B surface antibody; HBV: hepatitis B virus; HCV: hepatitis C virus; Na: sodium; Plt: platelet count; K: potassium; PT-INR: prothrombin time and international normalized ratio (PT/INR); RBC: red blood cells; T-Bil: total bilirubin; WBC: white blood cells

Parameter	Results	Normal values
WBC (count per μL)	9,800	3,300-8,600
RBC (count per μL)	389×10^4^	435-555×10^4^
Hb (g/dL)	12.0	13.7-16.8
Plt (count per μL)	21.5×10^4^	15.8-34.8×10^4^
PT-INR	1.54	0.85-1.15
Alb (g/dL)	2.9	3.8-5.2
BUN (mg/dL)	17	8-20
Cre (mg/dL)	0.84	0.65-1.07
AST (U/L)	157	13-30
ALT (U/L)	19	10-42
LDH (U/L)	146	124–222
ALP (U/L)	177	38-113
γ-GTP (U/L)	423	13-64
T-Bil (mg/dL)	0.7	0.4-1.5
Na (mmol/L)	137	138-145
K (mmol/L	4.3	3.6-4.8
Cl (mmol/L)	103	101-108
CRP (mg/dL)	0.27	0-0.14
HBsAg	Positive	Negative
HBsAb	Positive	Negative
HBcAb	Positive	Negative
HBV-DNA	Negative	Negative
HCVAb	Negative	Negative

**Figure 1:**
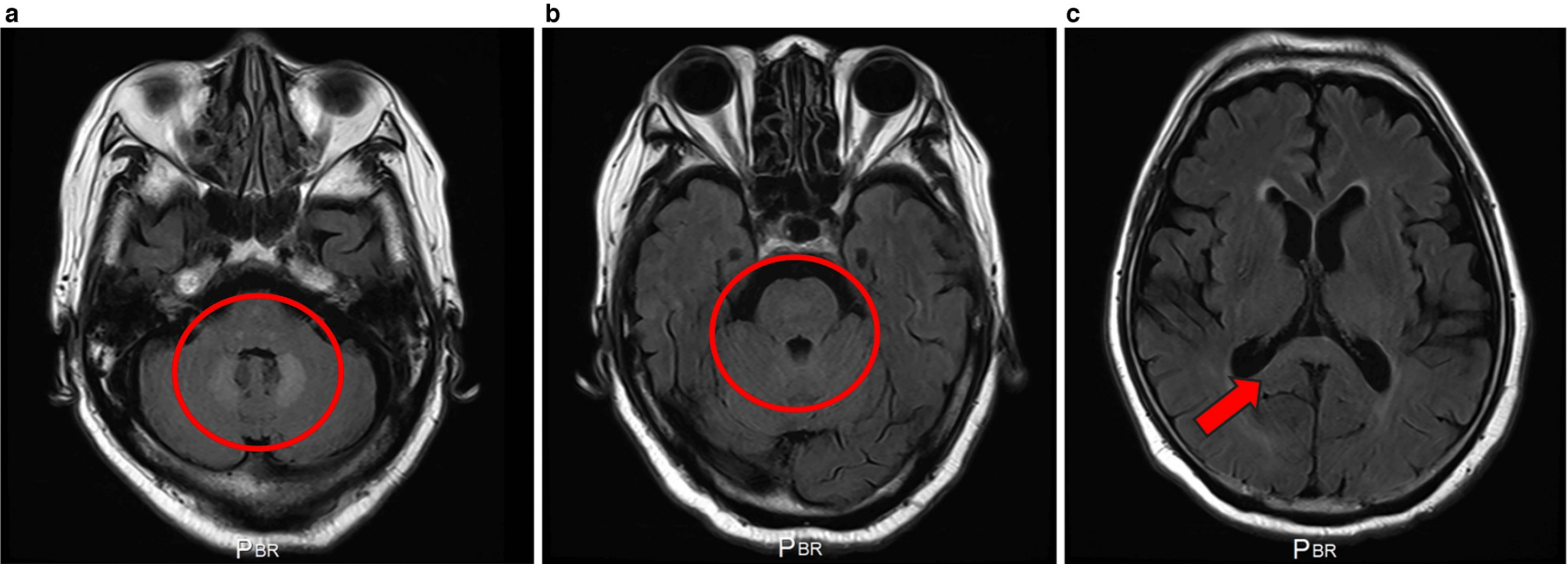
Initial brain MRI on day 63 of metronidazole therapy Axial FLAIR images show symmetric hyperintensities in the cerebellar dentate nuclei (a; red circles) and dorsal midbrain tegmentum (b; red circles), with a faint lesion in the splenium of the corpus callosum (c; single red arrow). The bilateral, posterior fossa-predominant distribution is characteristic of metronidazole-induced encephalopathy. FLAIR: fluid-attenuated inversion recovery; MRI: magnetic resonance imaging

Brain magnetic resonance imaging (MRI) on day 63 demonstrated bilateral symmetric T2/FLAIR hyperintensities in the cerebellar dentate nuclei without contrast enhancement, with additional subtle signal abnormality in the dorsal midbrain tegmentum and a faint lesion in the splenium of the corpus callosum. DWI showed mild hyperintensity with corresponding ADC values in the normal-to-high range. These findings, together with the exposure history, established a diagnosis of MIE; metronidazole was immediately discontinued (Figure 1).

**Figure 2:**
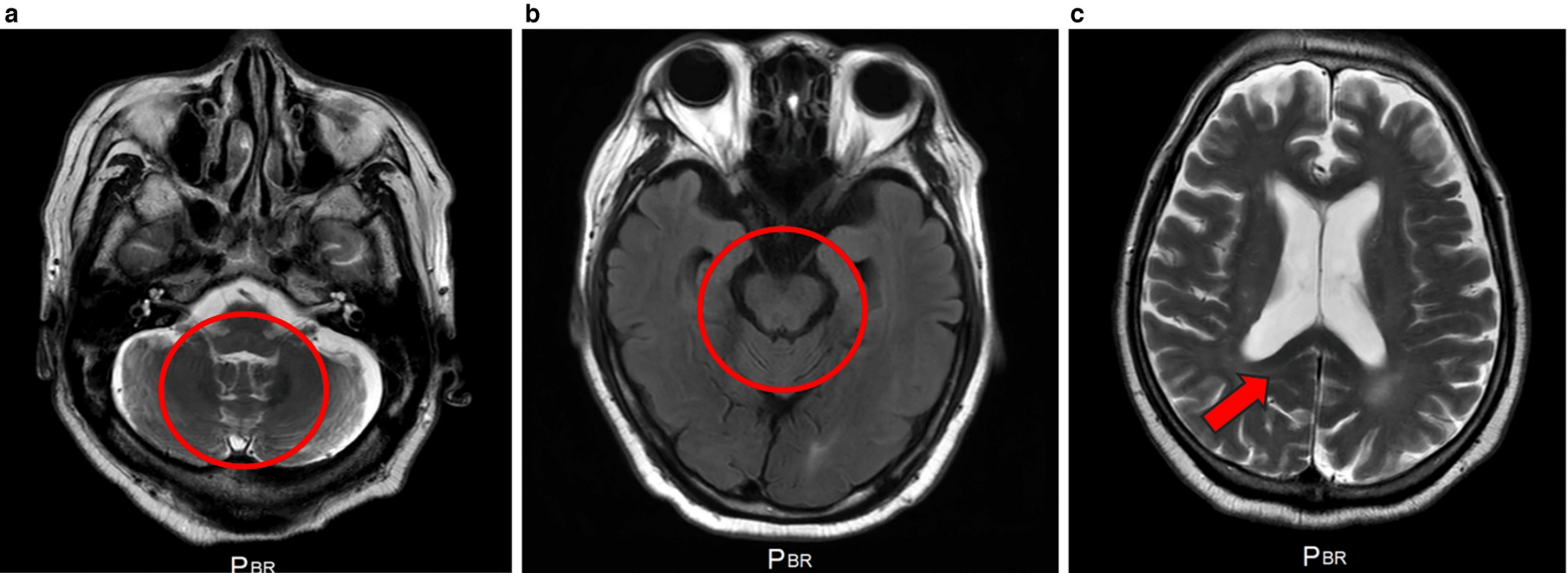
Follow-up brain MRI on day 92 after the discontinuation of metronidazole Axial FLAIR images (a, b; red circles) and an axial T2-weighted image (c; red arrow) demonstrate complete resolution of the previously observed symmetric hyperintensities in the cerebellar dentate nuclei and dorsal midbrain tegmentum, with the disappearance of the faint splenial lesion in the corpus callosum. The radiologic reversibility parallels the patient's clinical recovery and is characteristic of metronidazole-induced encephalopathy. FLAIR: fluid-attenuated inversion recovery; MRI: magnetic resonance imaging

On day 64, gait and postural instability transiently worsened, after which the patient's neurological symptoms gradually improved with supportive care consisting of intravenous fluids, nutritional support, and rehabilitation. Follow-up MRI on day 92 showed complete radiological resolution of the dentate-nucleus and associated lesions (Figure 2). By then, dysarthria and ataxia had markedly improved, and the patient was ambulating independently. The total cumulative metronidazole dose was 76.5 g (<100 g). Clindamycin was used as an alternative agent for ongoing anaerobic coverage thereafter, and no further neurological events occurred.

## Discussion

This case illustrates that MIE can occur at cumulative exposures <100 g and that re-exposure may precipitate rapid neurological worsening despite prior improvement, consistent with previous reports highlighting wide inter-individual variability in exposure and onset and emphasizing host susceptibility over simple dose dependence [1-3,7]. Among predisposing factors, cirrhosis is particularly relevant; reduced hepatic reserve lowers systemic clearance and prolongs the half-life of metronidazole and its metabolites, thereby increasing effective central nervous system exposure and lowering the clinical threshold for neurotoxicity [1,9,11]. Case reports and series document MIE at comparatively low cumulative doses in patients with hepatic dysfunction, including viral- or alcohol-related cirrhosis [7,8].

From a neuroradiological standpoint, symmetric dentate-nucleus hyperintensities on T2/FLAIR constitute the core imaging signature, with frequent additional lesions in the brainstem tegmentum and splenium of the corpus callosum [2,3,6]. DWI hyperintensity with normal-to-increased ADC, reflecting T2 shine-through, supports vasogenic edema as the dominant pathology, although ADC reduction, indicating cytotoxic edema, has been described in severe cases and may relate to worse clinical presentations [2,3,6,7]. Our patient's non-enhancing, symmetric dentate-nucleus lesions with DWI/ADC patterns congruent with vasogenic edema, together with the exposure history and clinical reversibility, were diagnostic.

The differential diagnosis includes Wernicke encephalopathy, posterior circulation stroke, central pontine myelinolysis, demyelinating disorders, and drug-induced encephalopathies such as cycloserine- or isoniazid-related toxicity [3,5,7]. In practice, the triad of temporal association with metronidazole, characteristic symmetric distribution, and reversibility after withdrawal is diagnostically decisive [1-5,7].

Disease management hinges on the immediate discontinuation of metronidazole. Most patients improve clinically within days to two weeks, with radiologic normalization lagging; corticosteroids have been used in isolated reports of severe or progressive cases but are not standard therapy [1,3,4,7]. For patients at risk for whom prolonged metronidazole therapy is anticipated, especially those with hepatic or renal dysfunction or prior exposure, pragmatic safeguards include baseline neurological assessment, re-evaluation after 4-6 weeks or with an increasing cumulative dose, and prompt drug withdrawal with early MRI at the first sign of cerebellar dysfunction; where microbiologically acceptable, clindamycin or other alternatives should be preferred to avoid rechallenge [10].

## Conclusions

The threshold for metronidazole neurotoxicity appears to be lower in patients with cirrhosis, and MIE may occur at cumulative doses <100 g. New-onset cerebellar signs during metronidazole therapy should prompt immediate discontinuation, early MRI, and avoidance of re-exposure. Additionally, re-exposure should be avoided, and routine tracking of the cumulative dose with regular bedside cerebellar screening may be adopted to prevent persistent deficits.
